# Overcoming Acquired MET-Driven Resistance to First-Line Lorlatinib: Successful Combination of Lorlatinib and Envafolimab in an ALK-Positive NSCLC Patient with Ultra-High PD-L1 Expression

**DOI:** 10.3390/curroncol33050258

**Published:** 2026-04-29

**Authors:** Lu Ding, Reyizha Nuersulitan, Jingjing Wang, Hanxiao Chen, Minglei Zhuo

**Affiliations:** Key Laboratory of Carcinogenesis and Translational Research (Ministry of Education), Department I of Thoracic Oncology, Peking University Cancer Hospital & Institute, Beijing 100142, China; 2511110737@bjmu.edu.cn (L.D.); 2365610852@bjmu.edu.cn (R.N.); 1765610740@bjmu.edu.cn (J.W.); 0065610713@pku.edu.cn (H.C.)

**Keywords:** ALK-positive non-small cell lung cancer, lorlatinib, envafolimab, TKI resistance, high PD-L1 expression, case report

## Abstract

Anaplastic lymphoma kinase (ALK) rearrangement is a well-established oncogenic driver alteration in non-small cell lung cancer (NSCLC), and ALK tyrosine kinase inhibitors (TKIs), particularly lorlatinib, have significantly improved the prognosis of ALK-positive NSCLC patients. Although high programmed death-ligand 1 (PD-L1) expression (≥50%) is generally associated with favorable responses to immune checkpoint inhibitors (ICIs), PD-L1 has not been shown to reliably predict ICI benefit in ALK-rearranged disease, and optimal management after ALK TKI resistance remains challenging. Herein, we report a case of an elderly patient with ALK-rearrangement and exceptionally high PD-L1 expression (TPS ≥ 95%) NSCLC who experienced disease progression following first-line lorlatinib with genetically confirmed MET amplification. The patient subsequently received an exploratory combination of continued lorlatinib plus envafolimab and achieved partial response (PR) with manageable tolerability after 4 months, highlighting a potential sequential strategy that may warrant further investigation in select ALK-positive NSCLC patients exhibiting both bypass pathway activation and exceptionally high PD-L1 expression.

## Simple Summary

Targeted therapy has significantly improved outcomes for patients with anaplastic lymphoma kinase (ALK)-positive non-small cell lung cancer (NSCLC); however, the emergence of resistance remains an inevitable clinical challenge. Standard treatment after progression on third-generation ALK tyrosine kinase inhibitors (TKIs) is often restricted to chemotherapy, which may not be suitable or acceptable for all patients. We describe a patient with ALK-positive NSCLC and ultra-high PD-L1 expression whose disease progressed after first-line lorlatinib treatment. A repeat biopsy identified acquired MET amplification as a potential resistance mechanism. Given the limited standard options, the patient received a personalized combination of continued lorlatinib, envafolimab (an anti-PD-L1 antibody), and localized radiotherapy. This regimen induced a significant radiological response with a manageable safety profile. This case demonstrates that, in highly selected patients, a multi-modality strategy guided by repeat biopsy and biomarker profiling may provide meaningful clinical benefit after resistance to targeted therapies.

## 1. Introduction

As crucial oncogenic drivers, anaplastic lymphoma kinase (ALK) translocations account for 3–7% of non-small cell lung cancer (NSCLC) cases, exhibiting a strong predilection for younger never-smokers with adenocarcinoma [1]. The predominant variant, echinoderm microtubule-binding protein 4 (EML4-ALK) fusion, constitutively activates downstream pathways such as PI3K/AKT and RAS/MEK/ERK, promoting tumor cell proliferation [2]. Since the approval of the first-generation ALK tyrosine kinase inhibitor (TKI) crizotinib in 2011, the treatment landscape for ALK-positive NSCLC has been revolutionized. As of 31 December 2024, the U.S. Food and Drug Administration (FDA) has approved six ALK TKIs for clinical practice. These include the first-generation crizotinib; the second-generation alectinib, brigatinib, ceritinib and ensartinib; and the third-generation lorlatinib.

Among these, lorlatinib-a third-generation ALK TKI, demonstrates potent antitumor efficacy and superior blood–brain barrier penetrability due to its unique macrocyclic amide architecture. Data from the Phase III trial CROWN demonstrated that lorlatinib achieves a 5-year progression-free survival (PFS) rate of up to 60% and a 5-year intracranial progression-free rate of up to 92% [3]. In subgroup analyses, the Asian population—including Chinese patients—showed particularly favorable outcomes, with median PFS not yet reached after more than 5 years of follow-up and 5-year PFS rates exceeding 63% overall and approximately 70% in Chinese patients, underscoring the durability of response in these cohorts [4]. Despite the remarkable efficacy of ALK TKIs, acquired resistance to targeted therapies remains an inevitable challenge [5]. Treatment strategies post-lorlatinib progression are not well-defined, especially in cases where resistance develops following first-line use with complex molecular profiles. While pemetrexed-based chemotherapy and clinical trials remain primary options for subsequent-line treatment, their outcomes remain suboptimal. Consequently, exploring effective strategies to overcome lorlatinib resistance remains a significant research priority in the field of ALK-positive NSCLC.

Over the past few decades, Immune checkpoint inhibitors (ICIs) targeting the PD-1/PD-L1 axis and CTLA-4 have recently redefined the standard of care for advanced, driver-negative NSCLC. PD-L1 expression levels typically correlate positively with the efficacy of immunotherapy [6]. Retrospective studies indicate that a significant proportion of patients with ALK-positive NSCLC present with PD-L1 positivity [7]. However, previous clinical studies indicate that the ALK-positive NSCLC patient population derives minimal benefit from monotherapy with ICI. Given the limited efficacy of chemotherapy after the development of resistance to ALK TKIs, combining ALK TKIs with ICIs was considered a promising therapeutic strategy. Nevertheless, multiple clinical trials investigating ALK TKIs combined with PD-1/PD-L1 ICIs (e.g., JAVELIN Lung 101, CHECKMATE 370) were prematurely terminated due to Grade 3–4 hepatic toxicity, posing significant hurdles to this approach [8,9].

With growing insights into the toxicity mechanisms underlying this combination strategy and the emergence of novel drugs with unique pharmacokinetic profiles, re-evaluating safe and effective combination regimens is warranted. Herein, we present the case of a patient with ALK-positive NSCLC and ultra-high PD-L1 expression (TPS ≥ 95%) who experienced disease progression following first-line lorlatinib with acquired MET amplification. Subsequently, the patient achieved a partial response (PR) after 4 months of combination therapy with lorlatinib and envafolimab. As the world’s first approved subcutaneous PD-L1 inhibitor, envafolimab offers advantages in its route of administration and presents a distinct safety profile compared to traditional intravenous agents. Through this case report, we aim to provide preliminary insights into the efficacy and safety of combining a third-generation ALK TKI with a novel PD-L1 inhibitor in the specific setting of lorlatinib-resistant, ultra-high PD-L1 ALK-positive NSCLC. Our findings provide preliminary clinical evidence and highlight a potential therapeutic strategy for this challenging patient population.

## 2. Case Presentation

**Figure 1:**
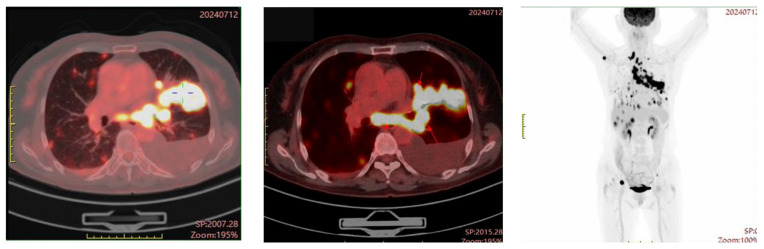
PET-CT at diagnosis. The red arrows indicate the hypermetabolic primary tumor in the left lung and the metastatic lesions.

In July 2024, a 69-year-old female patient with no smoking history was admitted to the Department of Thoracic Medical Oncology at Peking University Cancer Hospital with a productive cough for two months. Chest computed tomography (CT) revealed a mass in left lung, and pathological evaluation of a CT-guided biopsy sample confirmed lung adenocarcinoma. Immunohistochemistry (IHC) analysis showed CK5/6(−), Napsin A(+), P40(−), pan-TRK(−), PD-L1(22C3)(TPS > 95%), ALK-Ventana(+), Cmet(−). Positron emission tomography-CT (PET-CT) identified a hypermetabolic mass in the lingular segment of the left upper lobe (SUVmax 21.3), measuring approximately 6.3 × 3.6 × 3.8 cm (Figure 1). Extensive metastatic involvement was observed, including bilateral supraclavicular, mediastinal, and hilar lymph nodes; multiple bilateral pulmonary and pleural metastases with bilateral pleural effusion; as well as distant metastases in the liver, pancreatic body, bilateral adrenal glands, and multiple bones (Figure 1). Additionally, cranial magnetic resonance imaging (MRI) detected a 4-mm enhancing nodule in the left parietal lobe with surrounding edema. Based on these findings, the patient was diagnosed with stage IVB lung adenocarcinoma (cT4N3M1c).

**Figure 2:**
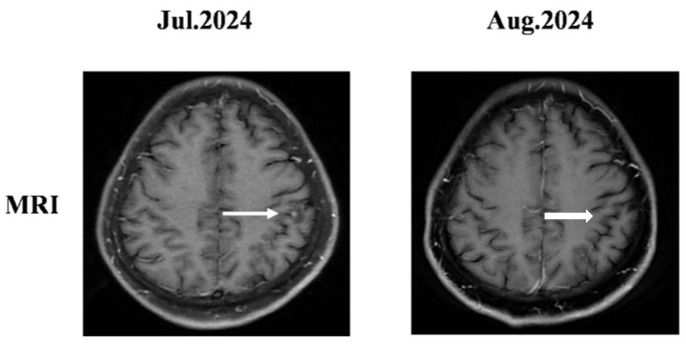
After one month of lorlatinib treatment, the cranial MRI indicated that the metastatic lesion in the left frontal lobe had disappeared. The white arrows indicate the brain metastases.

Given the presence of brain metastases and the ALK-positive status confirmed by IHC, lorlatinib was initiated as first-line therapy (100 mg QD) on 17 July 2024, due to its superior blood–brain barrier penetrability and robust systemic control. Follow-up assessment revealed significant shrinkage of the primary lung lesion and mediastinal lymph nodes, along with complete resolution of the brain metastases (Figure 2). The patient achieved a PR and remained stable during subsequent follow-ups.

**Figure 3:**
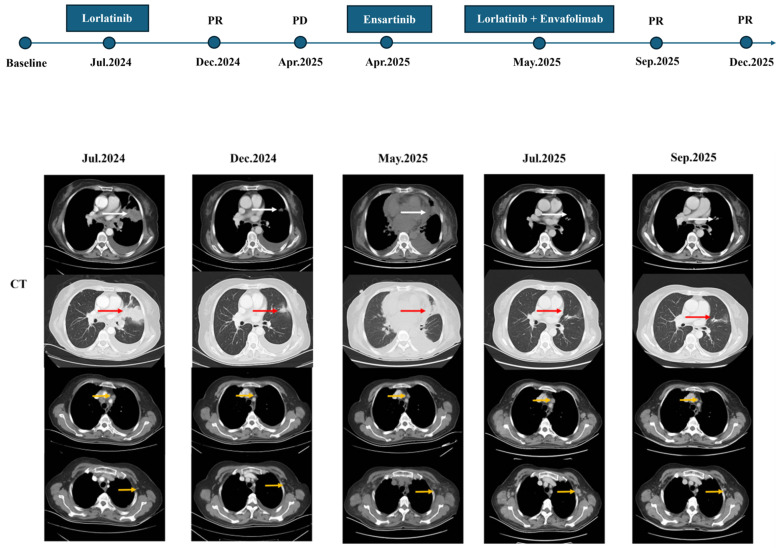
Time course depicting treatment process and radiological evaluation. Following lorlatinib therapy, the primary pulmonary lesion and mediastinal lymph nodes decreased in size; pleural effusion reduced. After 9 months of lorlatinib treatment, pericardial effusion and pleural effusion increased, axillary lymph nodes enlarged, and the disease progressed. Following treatment with lorlatinib combined with envafolimab, the primary pulmonary lesion decreased in size compared to baseline, pericardial effusion and pleural effusion reduced, and axillary lymph nodes shrank. The white arrows indicate the primary lesion in the mediastinal window, the red arrows indicate the primary lesion in the lung window, and the orange arrows indicate the lymph node metastases.

**Figure 4:**
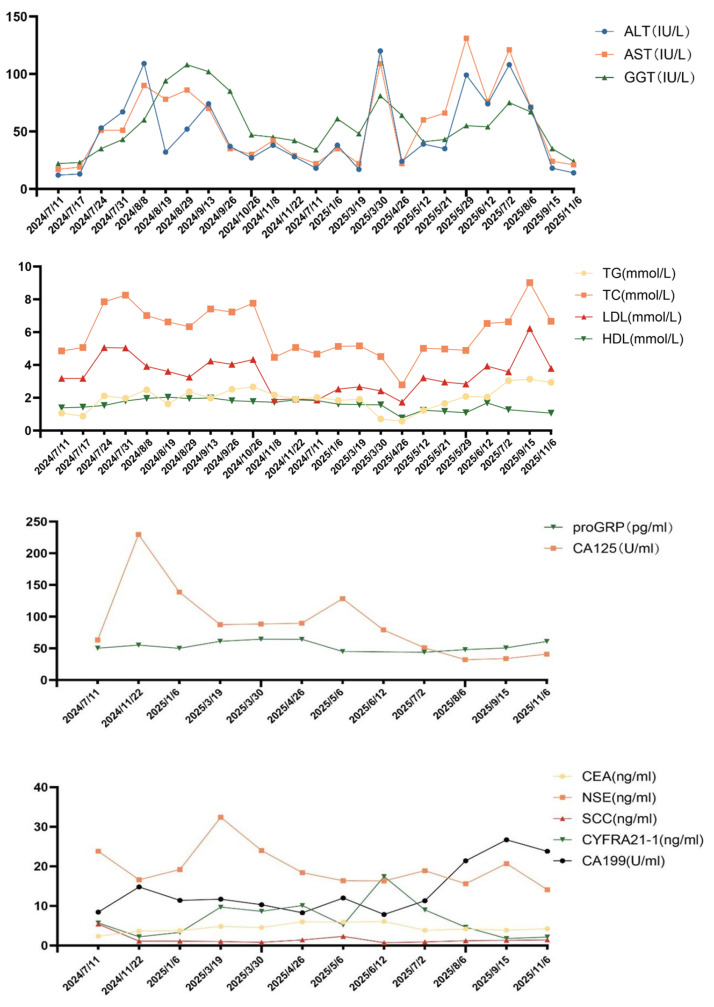
Trends of liver function-related indicators, lipid levels and tumor markers during treatment.

However, in March 2025, the patient developed left lower limb pain. Lumbar MRI with diffusion-weighted imaging (DWI) revealed soft tissue lesions involving the L3 vertebra as well as the right ilium, ischium, and pubis, suggestive of malignancy. Concurrent chest and abdominal CT scans indicated a new nodule in the right upper lung lobe and enlargement of multiple lymph nodes in the mediastinum, hila, and right supraclavicular region. In April 2025, CT-guided inguinal lymph node biopsy pathology confirmed metastatic lung adenocarcinoma. IHC showed ALK-Ventana(+), PD-L1(22C3)(TPS = 95%), and strong c-Met positivity (95%). Next-generation sequencing (NGS) identified a TP53 p.Y126H mutation (29.1%), MET amplification (5.3-fold), an EML4-ALK fusion (EX17:EX20, 19.8%), and CDK4 amplification (1.9-fold) (Table S1). Chest and abdominal CT revealed disease progression, characterized by enlargement of lymph nodes in the mediastinum, bilateral hila, right supraclavicular region, and left axilla. Furthermore, CT showed increased left-sided pleural effusion, new-onset right pleural effusion, increased pericardial effusion, and enlarged lymph nodes along the right external iliac vessel. Following the confirmation of disease progression, an interim trial of ensartinib was attempted. Unfortunately, this treatment was terminated after just two weeks due to the onset of cardiac failure and arrhythmia (Table S2). In light of this intolerance, persistently ultra-high PD-L1 expression, and post-progression biopsy confirming MET amplification—along with the patient’s strong refusal of chemotherapy—a multidisciplinary team recommended a second-line regimen of lorlatinib (100 mg QD) combined with envafolimab (300 mg Q3W) on 22 May 2025. Palliative radiotherapy was administered in June 2025 to relieve pain: pelvic soft tissue metastases (95% PTV1) received 40 Gy/10 fractions, while right iliac para-aortic lymph nodes and bone metastases (95% PTV2) received 30 Gy/10 fractions. Concurrently, thoracentesis and drainage were performed to manage the pleural effusion. This combination regimen demonstrated encouraging efficacy. After two months, follow-up CT showed the mass in the left upper lobe had significantly shrunk from 17 × 8 mm to fibrotic streaks, and the bilateral pleural and pericardial effusions had completely resolved. After four months, multiple diffuse pulmonary nodules had decreased in size from 7 × 5 mm to 6 × 5 mm, the left axillary lymph node reduced from 21 × 15 mm to 12 × 9 mm, and the right external iliac nodes regressed from 14 × 9 mm to 11 × 8 mm (Figure 3). Tumor markers also declined. Treatment-related adverse events included Grade 2 hepatic dysfunction and hyperlipidemia, both of which were successfully managed with hepatoprotective agents (diammonium glycyrrhizinate, silymarin, and ursodeoxycholic acid) and lipid-lowering therapies (pravastatin and evolocumab). Following these interventions and a brief 2-week suspension of envafolimab, liver function recovered, and lipid levels stabilized without further elevation (Figure 4). At the time of this report, the patient remains on the combination regimen with ongoing clinical and radiologic follow-up.

## 3. Discussion

This case report details an elderly female patient with EML4-ALK fusion characterized by exceptionally high PD-L1 expression and an extensive tumor burden at initial diagnosis. The patient achieved a PR to first-line lorlatinib but developed systemic progression approximately 9 months after treatment initiation. Re-biopsy and NGS demonstrated MET amplification, supporting an off-target bypass resistance mechanism. The patient explicitly declined chemotherapy. Ultimately, a combination therapy comprising lorlatinib, the PD-L1 inhibitor envafolimab, and local radiotherapy achieved a second partial response. This case offers a novel therapeutic perspective for managing ALK-positive NSCLC patients who develop resistance characterized by high PD-L1 expression and secondary MET amplification.

The patient experienced disease progression 9 months after initiating first-line lorlatinib, which is a shorter PFS than the long-term benefit observed in the CROWN trial. However, exploratory data presented at the 2024 World Conference on Lung Cancer suggest that patients with early progression on frontline lorlatinib (≤12 months) more frequently had unconfirmed baseline ALK positivity, detected TP53 mutations, and a greater baseline tumor burden than those who remained progression-free beyond 5 years [10]. In our case, baseline NGS was not performed, and the initial diagnosis relied solely on ALK-Ventana IHC, precluding a direct comparison between the baseline and post-progression molecular profiles. Nevertheless, the relatively early progression at 9 months, the extensive baseline tumor burden, and the TP53 mutation detected at progression raise the possibility that this patient harbored high-risk biological features at baseline, which may have contributed to the earlier failure of first-line lorlatinib. Previous studies by Nie et al. suggest that high PD-L1 expression (≥50%) correlates with poorer PFS in ALK-positive NSCLC patients treated with ALK TKI [11]. The ultra-high PD-L1 expression (TPS ≥ 95%) and massive systemic tumor burden characterized a highly aggressive biological profile. Acquired resistance mechanisms to lorlatinib can be categorized into on-target and off-target pathways [12]. Lorlatinib is a highly potent third-generation TKI with broad coverage against ALK domain mutations. Clinical evidence from the CROWN study and subsequent resistance profiling indicates that acquired resistance is predominantly driven by off-target bypass mechanisms, while emergent on-target ALK resistance mutations were not detected in the available end-of-treatment ctDNA analyses [3]. In this patient, the identification of MET amplification represents a classic off-target bypass activation mechanism. MET gene amplification leads to non-HGF-dependent MET receptor phosphorylation and sustained activation of downstream signaling (e.g., PI3K/AKT and MAPK) [13]. Thus, even with sustained ALK inhibition by lorlatinib, tumor cells can maintain survival and proliferation via the MET pathway. The patient’s secondary biopsy provided critical evidence for subsequent treatment decisions.

Previous studies have established that PD-L1 positivity (TPS ≥ 1%) occurs in approximately 59.8% of ALK-positive NSCLC patients, with 19.2% exhibiting high PD-L1 expression (TPS ≥ 50%) [14]. This may be associated with ALK fusion protein-activated signaling pathways involving signal transducer and activator of transcription 3 (STAT3) and hypoxia-inducible factor 1-alpha (HIF-1α), which upregulate PD-L1 expression [15]. However, while immune checkpoint inhibitors (ICIs) have become the standard of care for driver-negative NSCLC, patients with ALK-positive disease derive minimal benefit from ICI monotherapy. Notably, data from the IMMUNOTARGET registry demonstrated a 0% response rate to ICIs for patients with ALK fusions [16]. A retrospective study from Japan, including 190 patients, demonstrated that among 7 ALK-positive patients treated with pembrolizumab or nivolumab, the median progression-free survival (PFS) was a dismal 0.6 months (95% CI: 0.2–2.1), with all seven patients experiencing disease progression within three months [17]. This phenomenon is closely related to tumor microenvironment characteristics. ALK-positive NSCLC is generally characterized as a “cold” tumor type, featuring a low tumor mutational burden (TMB) and a paucity of tumor-infiltrating lymphocytes (TILs) [18], rendering it unresponsive to ICIs. Moreover, multiple clinical trials exploring the combination of ICIs and ALK TKIs were terminated prematurely due to severe hepatotoxicity. Consequently, the application of ICIs in this population requires rigorous evaluation and caution.

Regarding treatment post-lorlatinib progression, pemetrexed/platinum-based chemotherapy remains an effective standard palliative option [19]. In our patient, acquired MET amplification made combined ALK/MET inhibition a biologically rational strategy. As another approved ALK TKI with activity against MET, ensartinib was briefly attempted in clinical practice [20]. However, it was discontinued after two weeks because of cardiac failure and arrhythmia, and the patient also had clinically significant pleural and pericardial effusions, raising concerns about the tolerability of further TKI intensification. In addition, the patient firmly declined chemotherapy. Under these constraints, we reconsidered the post-progression biopsy findings, which showed persistently ultra-high PD-L1 expression together with ongoing ALK fusion, and this provided a rationale for cautiously exploring a regimen combining continued lorlatinib with PD-L1 blockade. When designing this regimen, safety was paramount. JAVELIN Lung 101 data showed lower liver toxicity rates in the lorlatinib plus PD-L1 inhibitor group compared to crizotinib plus PD-L1 inhibitor (7% vs. 17%) [8]. Envafolimab is a humanized camel-derived single-domain anti-PD-L1 antibody engineered with a human immunoglobulin Fc fragment fusion, reducing antibody-dependent cellular cytotoxicity (ADCC) and complement-dependent cytotoxicity (CDC) [21], potentially offering a distinct safety profile compared to conventional intravenous PD-1 inhibitors. Finally, the subcutaneous injection route offers simplicity and convenience, addressing the needs of elderly patients.

In the absence of preclinical or translational studies, the mechanisms underlying the response observed in this case remain speculative. We hypothesize that the efficacy stems from a synergistic remodeling of the tumor microenvironment (TME). Continued ALK inhibition by lorlatinib maintains suppression of the dominant driver pathway, and may additionally promote immunogenic cell death (ICD) and help reverse T-cell exhaustion [22]. This remodeling of the TME laid the groundwork for the subsequent anti-tumor effects of envafolimab. Furthermore, palliative radiotherapy may have provided a synergistic effect. As suggested by the KEYNOTE-001 trial, prior radiation can function as an immunological adjuvant to enhance subsequent immunotherapy outcomes [23]. This synergy potentially drove the observed abscopal-like efficacy [24]. However, because that study was not conducted in an ALK-rearranged population and did not evaluate lorlatinib combinations, the relative contributions of the TKI, PD-L1 blockade, and radiotherapy cannot be fully disentangled in this single case. Instead, it is highly probable that these three therapeutic modalities acted in concert to achieve the observed clinical benefit.

Regarding safety, adverse events (AEs) observed during treatment included Grade II hepatotoxicity and hyperlipidemia, consistent with known safety profiles. No serious AEs requiring permanent discontinuation occurred. Previous studies suggest that PD-L1 inhibitors generally exhibit a lower incidence of immune-related adverse events (irAEs) compared to PD-1 inhibitors [25]. As previously noted, this favorable tolerability is likely supported by envafolimab’s unique molecular structure, which reduces immune-mediated tissue damage [21]. The successful management of this patient through regular monitoring and timely intervention suggests that under the strict supervision of a multidisciplinary team (MDT), this combination represents a feasible and manageable strategy for selected patients.

Despite the striking clinical benefit observed in this patient, the combination of ALK TKIs and ICIs must be approached with extreme caution. Historically, early-phase trials evaluating the concurrent initiation of TKIs and PD-(L)1 inhibitors have reported unacceptably high rates of severe adverse events, predominantly hepatotoxicity and interstitial lung disease (ILD). In the present case, rather than concurrent initiation, envafolimab was added sequentially to a well-tolerated baseline of lorlatinib therapy. This staggered approach, coupled with rigorous clinical monitoring and the prompt management of emerging side effects, likely contributed to the favorable and manageable safety profile observed. We also acknowledge several limitations in our report. As this is a single-case observation, individual variations cannot be excluded, meaning the generalizability of the conclusions requires validation in larger cohorts. In addition, baseline NGS was not performed, which limited reconstruction of the full molecular evolution of resistance and precluded confirmation of baseline co-mutations or fusion subtype. Furthermore, our assessment remained at the clinical level without in-depth exploration of the underlying molecular mechanisms. Finally, given that the follow-up period was relatively short, whether this combined therapeutic regimen can yield long-term survival benefits still requires further surveillance.

## 4. Conclusions

In summary, we report a case of an elderly patient with ultra-high PD-L1 expression (TPS ≥ 95%) and ALK-positive NSCLC who achieved a sustained PR through a multimodal approach. This strategy, involving continued lorlatinib combined with envafolimab and radiotherapy, successfully addressed disease progression driven by acquired MET amplification after first-line lorlatinib treatment. To our knowledge, the patient remains on treatment with ongoing clinical benefit. This case provides a novel therapeutic approach for ALK-positive NSCLC patients who acquired resistance to ALK TKI. Specifically, with rigorous selection based on biomarkers such as exceptionally high PD-L1 expression and MET amplification, the combination of ALK-TKI and PD-L1 inhibitor may serve as a promising therapeutic strategy. While patients with oncogenic driver mutations are typically considered poor candidates for ICIs, this case suggests that, under specific resistance mechanisms and biomarker contexts, exceptions may exist that warrant further investigation.

## Data Availability

The data presented in this study are available on request from the corresponding author due to patient privacy and confidentiality concerns.

## Supplementary Materials

The following supporting information can be downloaded at https://www.mdpi.com/article/10.3390/curroncol33050258/s1. Table S1: Next-Generation Sequencing (NGS) Genomic Profiling at Post-Progression Biopsy; Table S2: Cardiac Biomarker and Clinical Dynamics Associated with ensartinib Treatment.
