# Supplementary material for: Overcoming Acquired MET-Driven Resistance to First-Line Lorlatinib: Successful Combination of Lorlatinib and Envafolimab in an ALK-Positive NSCLC Patient with Ultra-High PD-L1 Expression
Source: Curr Oncol. 2026 Apr 29;33(5):258. doi: 10.3390/curroncol33050258 (PMC13206712; doi:10.3390/curroncol33050258)
Supplement: Supplementary file 1 [file curroncol-33-00258-s001.zip › curroncol-4235052-supplementary.pdf]

Table S1. Next-Generation Sequencing (NGS) Genomic Profiling at Post-Progression Biopsy

Gene	Alteration / Variant	Variant Allele Frequency (VAF) / Copy Number (CN)
ALK	EML4-ALK fusion (EX17:EX20)	VAF: 19.8%
MET	Amplification	CN: 5.3x
TP53	p.Y126H missense mutation	VAF: 29.1%
CDK4	Amplification	CN: 1.9x

Table S2. Cardiac Biomarker and Clinical Dynamics Associated with Ensartinib Treatment

Timepoint	NT-proBNP (pg/mL) Normal range: <300	Clinical Presentation& Intervention
Baseline	489	No signs of heart failure
After 2 weeks of ensartinib	3282	Acute dyspnea, and edema.Ensartinib immediately terminated and administered intravenous loop diuretics.
1 week after discontinuation	415	Symptoms resolved; cardiopulmonary status stabilized.
